# Chicago Medical Society

**Published:** 1884-03

**Authors:** 


### CHICAGO MEDICAL SOCIETY.

At the meeting of this society on the evening of January 7, 1884, Dr. J. G. Kiernan read a paper on "Perverted Sexual Instinct." Upon this topic the literature of the past was reviewed to a considerable extent by the writer, of which the following extracts have been taken, a part of the phraseology not being in very common use:

Casper was the first to call attention to the condition known as sexual perversion, but not until several works had been published by a Hanoverian lawyer, Ulrichs, himself a sufferer from the disease, did the matter become the subject of scientific study on the part of physicians. Westphal was the first to discuss it, and described two cases, one, a female, who, when a child, is said to have been fond of boy's games and of putting on male attire. From her eighth year she had felt drawn to certain girls and liked to express her love for them, kiss them, and induce them to let her touch their genitals. She came of a neurotic ancestry, and displayed somatic signs of degeneration; she had imperative conceptions, and displayed symptoms of insanity other than the perversion. His second case was a male, who felt attracted by males. Servals reported a case, that of a male, who in addition to sexual perversion was morally imbecile. Schminke and Scholz have reported cases in males. Westphal, in addition to the two cases above cited, has later reported an additional case, that of a male. Krafft Ebing, after citing four cases (all males), concludes, that the main symptoms of the condition are first congenital absence of normal sexual feeling, and at times repugnance to normal intercourse, although they appear sexually developed normally.

The sexual psychical phenomena are replaced by this perversion. Sexual desire appears early, and extends to the same sex, or at most, this feeling finds expression in mutual onanism, but as a rule, pæderasty is repugnant.

Tamassia and Legrand du Saulle have reported one case each —both males. Stork reports three cases; one displaying primary monomaniacal tendencies. Raggi has reported a case of what he calls sexual perversion in a male, who, from having enlarged breasts, created the delusion that he was a woman. Charcot and Mognan have reported one case. In this country, five contributions have been made to this subject; one by Dr. H—— (See anonymous contribution to New York *Medical Record*, April 18, 1881), who describes the case of a male; one by Spitzka, who deals with a historical case; one by Blumer, who describes in a very thorough manner the case of a male, in whom the perversion had very Platonic manifestations (a kind of love of which Plato was a great expounder). Shaw and Ferris reported one case, which, however, was not well observed; and one case by Wise, who describes the Lesbian loves (from Lesbos, an old Greek city) of two victims of this perversion.

The author has seen three cases, two of which were impure; the other, a female, aged 22, whom he describes as being of neurotic ancestry on the paternal side, her cranium and face being asymmetrical. The patient has always had a fondness for dressing in male attire, and she feels at times sexually attracted by some of her female friends, with whom she has indulged in mutual masturbation. These feelings come at regular periods, and are then powerfully excited by the sight of the female genitals. In the interval she manifests repugnance to attentions from men. She is aware of the fact that while her lascivious dreams and thoughts are excited by females, those of her female friends are excited by males. She regards her feeling as morbid. At times she is troubled by imperative conceptions, such as that if she turn her head around she will break her neck; in consequence, she carries her head in a very constrained position.

Regarding the cause.—Excluding the impure cases of Ulrich's, Dr. Kiernan stated that thus far there have been twenty-seven cases reported, *four females, the rest males*, which would seem to show that sex predisposed to the affection; but it should be remembered that a history of female sexual desire is not easily obtained, that in nearly every case a neurotic ancestral history was obtained, and that the condition then appeared to be congenital.

*Treatment.*—To remove this perversion is out of the question. Dr. Wise has recommended that all such cases be sent to an asylum. Krafft Ebing proposed that these patients be exempt from legal penalties and allowed to follow their inclinations when harmless and when not violating public modesty. The case recited by the author he treated as if it were one of nymphomania, and by antaphrodisiac measures. He sought to strengthen the patient's will at the same time by instituting a course of intellectual training. The patient has been aided by these means so far as to be liable to control her perverse inclinations.

Dr. E. Andrews discussed the paper by saying cases of this kind occurring to people living in cities situated in the southern half of Europe are not regarded as cases of insanity, and in Itaiy many boys open a vicious trade to travelers, which, no doubt, is due to vicious associations there. Among the Latins and Greeks, and people living along the Mediterranean Sea, vicious habits of all sorts were practised 2,000 years ago, according to some works on ancient literature. In a certain saloon parlor in Paris there is exhibited a fine painting of a nude figure to represent a hermaphrodite, for certain kinds of lascivious purposes, and both sexes view it. Prostitutes frequently are known to be affected with chancroids on the verge of the anus, and the men frequenting the place often contract specific disease in the mouth.

Dr. R. E. Starkweather inquired of the essayist if he considered the cases that the newspapers had published so much recently about, occurring in Wisconsin, a *mutual* or mental disease? Both the persons are said to be females, and the latest report says that one is pregnant.

Dr. Kiernan thinks there is scarcely a civilized nation in which the vice is not practised; and this vice aspect is common in Asia, also in Naples.

Among the Pueblo Indians, one of their number is delegated as a pæderast. He, however, thinks these cases should be divided into two classes, one of which are vicious, the other perverted. Regarding the last speaker's interrogatory, he thinks we can reject the pregnancy part of the question; and then, too, in the case he alludes to we are not assured that both are women, for the scrotum of a man may be adjusted so as to elude detection; and other things being equal, a male person may dress in female clothing, and readily pass as such.

The discussion on this topic was then supplemented by a well prepared paper by Dr. Charles E. Webster, On Pott's Disease, and who exhibited a mechanical appliance of his own invention, that he had manufactured for the relief and cure of dorsal caries. The apparatus was examined by the gentlemen present, and many of them endorsed it as being superior to the jury-mast and head-lift, it being far less cumbersome than the former, which is apparently always in the way. The paper is an original contribution, and differs somewhat from the literature upon this subject. A description of the apparatus and mode of application we cannot well illustrate without the aid of a cut of the same, and we are, therefore, obliged to omit giving the minutiæ of the invention, but take pleasure in giving some of the points of interest regarding the causes, pathology, and the mechanical problem in the treatment of chronic non-suppurative spondylitis. We will add, then, that the general health of the patient has been much impaired, whether it arises from a peculiar diathesis, or the result of the disease, although this may not be as pertinent to the present inquiry as the local causes which arise from irritation of various kinds. Thus, the motion of the heads of the ribs, the lateral, and back and forth motion between the vertebræ, and the pressure of the superincumbent parts, may add greatly to the crumbling of the spinal column; and this process, which lowers the head and shoulders, pressing the sternum forward and the spinous processes backward, continues until the capacity of the thorax is diminished, and curvature finally results. This condition progresses until the ribs reach the crest of the ilium, and the patient grows anæmic and emaciated, and ultimately the sad spectacle is that of deformity from the pathological process that has been going on until a patient will be presented to the orthœpœdist for treatment, for whom one of the first measures of importance consists in permitting him exercise in the open, invigorating air.

The indirect advantage of having the patient occupied by this pleasant pursuit is equally counterbalanced in a direct advantage to his general health, and if malnutrition or any other abnormal condition of the system improve, there is hope that the caries may be arrested. Good diet is also an adjuvant and *sine qua non* to the outdoor life. The capacity of the thorax, naturally diminished by the disease, is generally still more reduced by surgical (unwise surgical appliances) as we ordinarily see them adjusted. The motion of the heads of the ribs in inspiration undoubtedly acts as an irritant. This can be readily prevented by constricting the chest, but for the reason just stated is inadmissible ; on the contrary, it may even be necessary to encourage such motion at the expense of temporarily increased inflammation, for the sake of additional lung room. The prime factor in perpetuating the caries is downward pressure. This is constant while the body is in the vertical position, and it is therefore necessary to support the weight of the parts above the point of disease, to counterindicate the tendency of grinding out the bodies of his vertebræ, to overcome the crumbling spinal column and resist the toppling over in the direction of the least resistance.

The weight to be supported consists of the head, arms, and that portion of the trunk superior to the part of the spine which is diseased. The only part of the body affording a base of support for apparatus to sustain this weight is the pelvis, *i. e.*, in individuals whose hips are sufficiently flaring to give a proper base, and in this proposition lies the substance, as we are able to deduce it, in the treatment of advanced cases of Pott's disease, as promulgated in the paper. The apparatus constructed by the surgeon he has amply demonstrated by several years' trial, and its usefulness is superior to any of the braces heretofore spoken of. The cost is estimated to be from $25 to $50.

Upon motion, the society adjourned. L. H. M.

---

CHICAGO MEDICAL SOCIETY.

